# Learning at your brain’s rhythm: individualized entrainment boosts learning for perceptual decisions

**DOI:** 10.1093/cercor/bhac426

**Published:** 2022-11-09

**Authors:** Elizabeth Michael, Lorena Santamaria Covarrubias, Victoria Leong, Zoe Kourtzi

**Affiliations:** Department of Psychology, University of Cambridge, Downing St, Cambridge CB2 3EB, United Kingdom; Department of Psychology, University of Cambridge, Downing St, Cambridge CB2 3EB, United Kingdom; Department of Psychology, University of Cambridge, Downing St, Cambridge CB2 3EB, United Kingdom; Psychology, School of Social Sciences, Nanyang Technological University (NTU), Singapore 6398818, Singapore; Lee Kong Chian School of Medicine, NTU, Singapore 308232, Singapore; Department of Psychology, University of Cambridge, Downing St, Cambridge CB2 3EB, United Kingdom

**Keywords:** EEG, entrainment, learning, perceptual decisions, visual cortex

## Abstract

Training is known to improve our ability to make decisions when interacting in complex environments. However, individuals vary in their ability to learn new tasks and acquire new skills in different settings. Here, we test whether this variability in learning ability relates to individual brain oscillatory states. We use a visual flicker paradigm to entrain individuals at their own brain rhythm (i.e. peak alpha frequency) as measured by resting-state electroencephalography (EEG). We demonstrate that this individual frequency-matched brain entrainment results in faster learning in a visual identification task (i.e. detecting targets embedded in background clutter) compared to entrainment that does not match an individual’s alpha frequency. Further, we show that learning is specific to the phase relationship between the entraining flicker and the visual target stimulus. EEG during entrainment showed that individualized alpha entrainment boosts alpha power, induces phase alignment in the pre-stimulus period, and results in shorter latency of early visual evoked potentials, suggesting that brain entrainment facilitates early visual processing to support improved perceptual decisions. These findings suggest that individualized brain entrainment may boost perceptual learning by altering gain control mechanisms in the visual cortex, indicating a key role for individual neural oscillatory states in learning and brain plasticity.

Interpreting the plethora of information that we encounter in complex and dynamic environments places high demands on our sensory systems. Training is known to improve our ability to make perceptual judgements (e.g. detect targets in cluttered scenes), a process known as perceptual learning (Goldstone 1998). Yet, there is striking variability among individuals in their ability to take into account previous experience when making perceptual decisions (Saarinen and Levi 1995; Christian et al. 2015; Siegelman et al. 2017). Recent work suggests that individual variability in behavioral performance may relate to dynamic changes in brain states; that is, fluctuations in cortical excitability across time (Freyer et al. 2013; Sigala et al. 2014). Yet, our understanding of the brain states that underlie variability in learning ability across individuals and within the same individual across different contexts remains limited.

Brain states are closely associated with oscillations (Sigala et al. 2014) that are known to reflect cyclical change in the excitability of neural populations and gate the flow of neural activity within and across brain regions (Fries 2015, 2005). Oscillations within the alpha band (8–12 Hz) have been shown to relate to performance in perceptual tasks, with both amplitude and phase acting as gating mechanisms on perception (Hanslmayr et al. 2005; Sokoliuk and VanRullen 2016; Hülsdünker et al. 2018). In particular, decreased pre-stimulus alpha amplitude has been associated with increased detectability of near-threshold stimuli (Ergenoglu et al. 2004; Hanslmayr et al. 2007; Iemi et al. 2017). Further, previous work suggests a key role of alpha phase in perceptual judgements. In particular, briefly presented stimuli are more likely to be detected when they are aligned at particular phases of the alpha cycle (Busch et al. 2009; Mathewson et al. 2009; Wyart and Sergent 2009). Further, the likelihood of phosphenes after brain stimulation varies with alpha phase (Samaha et al. 2017), and alpha phase alignment at stimulus onset results in changes to the latency of early components of visual evoked responses (Mathewson et al. 2009; Hülsdünker et al. 2018).

Recently, rhythmic stimulation (e.g. repetitive sensory stimulation at alpha rate) has been used as an interventional tool to drive neural oscillatory activity at the stimulation frequency and test the link between alpha oscillations and behavioral performance (Mathewson et al. 2010; Spaak et al. 2014; Sokoliuk and VanRullen 2016; Chota and VanRullen 2019). In particular, this flicker-induced brain entrainment protocol has been shown to result in increased alpha power and phase alignment (Notbohm and Herrmann 2016). Entrainment protocols often target a fixed frequency across all participants (e.g. alpha band mean of 10 Hz). Yet, there is accumulating evidence that individual variability in peak alpha frequency is functionally relevant for perceptual processing. Although there is significant variation in peak alpha frequency *across* individuals, an individual’s peak alpha frequency has been suggested to relate to their personal rate of perceptual information processing. In particular, peak alpha frequency has been shown to be stable *within* individuals (Grandy et al. 2013; Barzegaran et al. 2017) and relate to the individual’s temporal window of integration for perceptual input (Samaha et al. 2015; Samaha and Postle 2015; Ronconi and Melcher 2017; Ronconi et al. 2018).

Here, we ask whether entraining the brain at individualized alpha frequencies boosts individual learning ability. We individualized the rhythmic visual stimulation to entrain oscillatory brain states (i.e. alpha oscillations) and test their role in individual learning ability in a visual discrimination task. In particular, we first measured alpha frequency oscillations using resting-state electroencephalography (EEG) for each individual participant. We then used a visual flicker paradigm to induce individualized alpha entrainment (i.e. flicker rate was matched to individual alpha peak frequency) during training on a visual discrimination task (i.e. identifying radial vs. concentric Glass patterns embedded in noise). We individualized manipulations of alpha entrainment frequency versus phase to dissociate their effect on perceptual learning ability. We hypothesized that entrainment at an individualized—compared to non-individualized—alpha frequency would boost learning ability. Further, we tested whether learning depends on the oscillatory phase of stimulus presentation (peak vs. trough) and whether entrainment frequency and phase interact to support improved performance due to training. We predicted that individualized alpha entrainment targeting the trough phase for stimulus delivery would boost learning, based on previous work showing that the trough phase of alpha oscillations is associated with stronger disinhibition (Busch et al. 2009; Mathewson et al. 2011, 2010; Fakche et al. 2022) that may facilitate visual target detection from clutter. Our results demonstrate that entrainment at an individualized alpha frequency results in faster learning. This improvement was evident when visual patterns were presented at the trough rather than the peak phase of the flicker-induced entrainment, suggesting that alpha phase gates visual processing. Further, using EEG during training, we demonstrate that flicker-induced entrainment drives alpha brain frequencies and alters early visual processing to boost learning for perceptual decisions.

## Materials and methods

### Participants

Participants (*n* = 100) were 18–35 years old (mean age: 23.6, SD = 4.3). Participants (*n* = 10) were excluded due to experimental (e.g. using incorrect response keys, self-reported intolerance of the visual flicker) or technical (*n* = 4) issues. Further, participants with low-quality EEG data due to excessive eye movements (*n* = 6) were excluded from further analyses. Data from a total of 80 participants (*n* = 20 per experimental group) were included in further analyses following these exclusions. All participants had normal or corrected-to-normal vision, did not receive any prescription medication, were naïve to the aim of the study, gave written informed consent, and received payment for their participation. The study was approved by the University of Cambridge Ethics Committee.

### Stimuli

**Fig. 1:**
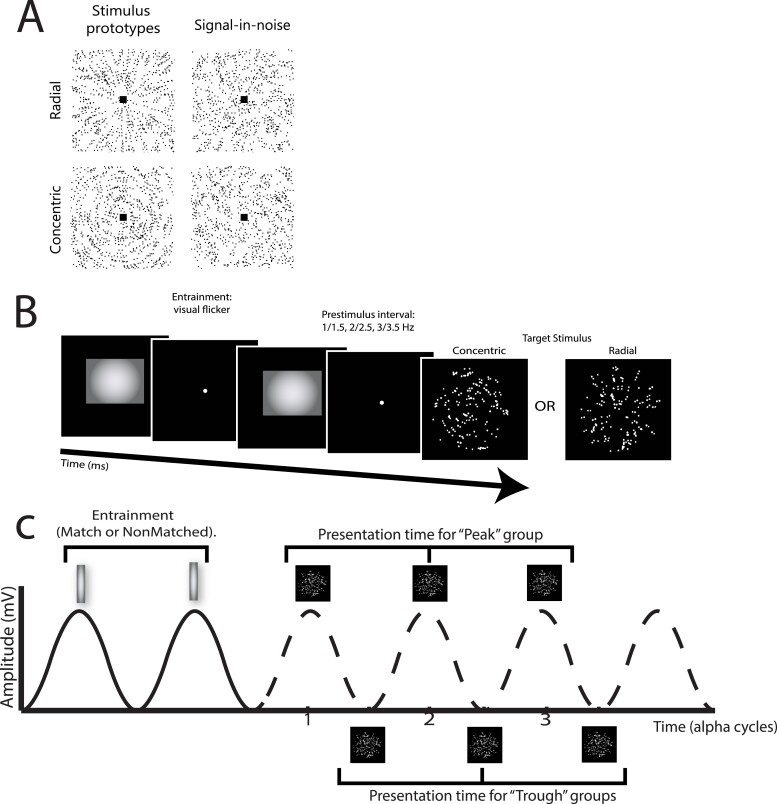
Experimental design and stimuli. A) Example stimuli comprising radial and concentric Glass patterns (stimuli are presented with inverted contrast for illustration purposes). Left: Prototype stimuli: 100% signal, spiral angle 0° for radial and 90° for concentric. Right: Stimuli used in the study: 25% signal, spiral angle 0° for radial and 90° for concentric. B) Trial design. Visual flicker (15 alpha cycles) was used to induce alpha entrainment. Each flash in the sequence was temporally separated by an interval equal to one cycle of each participant’s IAF. Following a blank interval at the end of the entrainment sequence (1–3 or 1.5–3.5 alpha cycles), the target stimulus was presented (200 ms). Participants were asked to judge whether the target stimulus was radial or concentric and indicated their decision with a button press. C) Experimental design. The entrainment frequency was either matched to the individual participant’s alpha frequency or was offset (nonMatched) by ±1 Hz. The onset of the target stimulus was set either at the peak or trough of the oscillation induced by the visual flicker by manipulating the interval after the entrainment sequence: for 10 Hz stimulation at the peak, the interval was 100, 200, or 300 ms; for 10 Hz stimulation at the trough, the interval was 150, 250, or 350 ms. These values were scaled according to the participant’s IAF. The solid line indicates the hypothesized trajectory of the entrained alpha oscillation during the visual flicker sequence. The dashed line reflects the hypothesized continuation of the entrained alpha oscillation after the flicker sequence has ended, with stimuli shown at all possible presentation times.

Stimuli comprised Glass patterns (Glass 1969) that were designed following parameters defined in previous studies (Pei et al. 2005; Frangou et al. 2018). Glass patterns were defined by white dot pairs (dipoles) displayed within a square aperture on a black background at the center of the screen (Fig. 1A). Stimuli were presented at visual angle of 7.9^o^ × 7.9^o^ (2.3 × 2.3 arc min^2^ per dot). Dot density was set to 3%, and the Glass shift (i.e. the distance between 2 dots in a dipole) was 16.2 arc min. For each dot dipole, the spiral angle was defined as the angle between the dot dipole orientation and the radius from the center of the dipole to the center of the stimulus aperture. The signal-to-noise ratio (SNR) was set to 24% ± 1% signal, that is, 24% of the dot dipoles were aligned according to the specified spiral angle (signal dipoles) for a given stimulus and the remaining dots were assigned a random orientation.

We generated radial (0^o^ spiral angle) and concentric (90^o^ spiral angle) Glass patterns by placing dipoles orthogonally (radial stimuli) or tangentially (concentric stimuli) to the circumference of a circle centered on the fixation dot. A new pattern was generated for each stimulus presented in a trial, resulting in stimuli that were locally jittered in their position. A small jitter (±1%–3%) was added to the SNR to control for low-level perceptual adaptation to local dot position and ensure that learning related to global shape rather than local stimulus features.

### Procedure

Each participant completed 2 experimental sessions on consecutive days (with the exception of one participant). In Session 1, participants practiced the pattern identification task for 50 trials followed by a resting-state EEG measurement (5 min) during which participants fixated on a central point on the screen (eyes open resting state measurement, EORS). Participants were then trained with flicker-induced entrainment (807 trials split in 4 blocks) on the pattern identification task with feedback. At the end of the training session, a second EEG EORS measurement was taken to test the reliability of the first peak alpha frequency measurement, as recent reports have reported a systematic shift in frequency over time (see Supplementary Material S1). On the following day (Session 2), participants completed 4 blocks (807 trials) without entrainment, feedback, or EEG. All participants were given the same instructions, task exposure, and presented with the different experiment phases (e.g. measuring resting state, practice tasks, main task) in the same order. They were encouraged to take short breaks between blocks to mitigate fatigue.

Entrainment was induced by flicker; that is, a high contrast square stimulus flickered (15 cycles flashes) at the center of the screen (at the same position as the Glass pattern stimuli) for a duration of (for 10 Hz stimulation) 1,500 ms at the beginning of each trial (Fig. 1B). Stimuli were generated using the Matlab toolbox, Psychtoolbox (Brainard and Vision 1997). Each flicker was designed as a visual “pulse,” with a high contrast white square displayed briefly followed by a blank interval. This inter-flash interval was set to 1 cycle of the selected alpha frequency for each participant depending on the experimental group they were allocated to. Although the 120 Hz refresh rate may limit the presentation rate, validation analyses suggest that the neural response targeted effectively the desired frequency (for validation of entrainment values, Supplementary Figs. S1.1–1.3).

Following the entrainment sequence, there was a brief interval before the stimulus onset. This interval was scaled according to the frequency of the entrainment and was randomly selected from 1 of 3 durations per trial: 1, 2, or 3 cycles (peak group), or 1.5, 2.5, or 3.5 cycles (trough groups). The test stimulus was then presented for 200 ms followed by a fixation point for 1.3 s. During this response period, participants were asked to indicate their judgment (radial vs. concentric pattern) by pressing 1 of 2 keyboard keys (left and right arrow keys). Visual feedback (green tick for correct response, red cross for incorrect response) was given at the same central location following the participant response (100 ms). A variable intertrial interval followed the feedback (mean 1.5 s with a uniform jitter of ±250 ms).

### Experimental groups

Participants were pseudorandomly allocated to 1 of 4 experimental groups defined by manipulations of the flicker-induced entrainment frequency and phase. First, the frequency of the entrainment flicker was either set to match the peak alpha frequency of each individual participant (“match”) or at ±1 Hz from the peak alpha frequency (“nonMatch”; +1 Hz: *n* = 11; −1 Hz: *n* = 9). Second, the phase alignment of the target stimulus (i.e. Glass pattern), with respect to the preceding flicker, was either at the “peak (P)” (the target appeared at whole-cycle intervals following the final flash) or “trough (T)” (target appears at half-cycle intervals following the final flash). This was instantiated by varying the number of cycles (1–3 for P, 1.5–3.5 for T) in the pre-stimulus interval (Fig. 1C). To test whether frequency effects were modulated by phase of stimulus delivery, we compared the 2 frequency-matched groups that differed in phase. However, for the nonMatch group, performance was assessed at the Trough phase only, as individual frequency-matched entrainment at the Trough phase was expected to show stronger behavioral improvement than entrainment at the Peak phase based on previous work (Busch et al. 2009; Mathewson et al. 2011, 2010; Fakche et al. 2022). These manipulations resulted in 3 experimental groups: T-Match (TM), T-nonMatch (TnM), and P-Match (PM). Finally, we tested an additional control group for the T-match group (random entrainment group); that is, we set the entrainment parameters to those of the T-match group based on individualized alpha frequencies but randomized the inter-flash intervals (keeping the total flicker duration the same across trials and groups). To avoid experimenter bias, we ensured that the researcher collecting the data did not have any information about baseline task performance before group allocation.

### E‌EG data acquisition

We measured EEG data for the 3 entrainment groups: T-Match, T-nonMatch, and P-Match groups. EEG data were acquired from 63 channels (BrainCap, Brain Products with 2 BrainAmp MR plus amplifiers), consisting of 61 scalp electrodes, arranged according to the extended 10–20 system (ground at AFz, reference electrode placed at FCz) and 2 bipolar channels for electrooculographic measures. One pair was placed horizontally, and another vertically around the left eye. We measured 3 EEG recordings in Session 1: resting-state EEG before training (EORS1), continuous EEG recording during training, and resting-state EEG after training (EORS2). For each resting-state EEG recording, we collected data (5 min) using the same BrainVision Recorder software as for the task EEG recording. For continuous EEG recording during training, we placed markers to indicate the onset of each flash in the flickering sequence and the target stimulus (Glass pattern) onset. Data were acquired with no additional online filter at a 1 kHz sampling rate. Caps were fitted with Ag/AgCl multitrode electrodes and filled with conductive gel. Impedances were kept at or below the recommended 5 kohm level.

### Behavioral data analysis

We calculated learning rate on performance across trials for each session. We fit a logarithmic function (*y* = *a* + b^*^log(*x*)), where *y* is the block accuracy) on mean accuracy values for each of 8 blocks (*n* = ~100 trials per block). To compare performance between the 2 sessions, we used the data from the final 2 blocks of the first session and the first 2 blocks of the second session. We estimated the slope (*b*) of the fits per session and compared this across intervention groups using a linear mixed effects model, where frequency (match/nonMatch) and phase (peak/trough) were used as predictors of learning rate. *T*-statistics for each predictor (or interaction) are reported, as a test for significance of the model coefficients. This approach is used to compare the 3 experimental groups accounting for the unbalanced design (i.e. for the nonMatch group, performance was assessed at the Trough phase but not the Peak phase). The Control group was compared to the T-Match group using a one-tailed independent group *t*-test, using an alpha level of 0.025 to account for multiple comparisons. In particular, we tested the hypothesis that learning is enhanced in the T-Match group in comparison with the Control group, as derived from the comparison of the 3 experimental groups.

### E‌EG resting-state analysis

We analyzed the resting-state EEG data with a pipeline that was adapted to allow timely estimates of individual alpha frequencies that were used to drive the entrainment protocol. Data were bandpass filtered (1–40 Hz) and re-referenced to the average reference. The continuous data were then divided into 10 s windows (10,000 samples). For each electrode in the occipital group (Oz, O1, O2, PO7, and PO8), a fast Fourier transform was applied to each window, using Matlab’s *fft* function.

The spectra were averaged across windows without smoothing to produce a single spectrum per participant. The individual alpha frequency (IAF) was defined as the peak between 8 and 12 Hz.

To compare the pre-training versus post-training peak alpha frequency, the same pipeline was applied to all resting-state data. To assess the stability of EORS, we compared the 2 measurements (EORS1 vs. EORS2) within and across groups (see Supplementary Material S1).

### E‌EG preprocessing

EEG data were preprocessed using routines from EEGLAB (Delorme and Makeig 2004) and custom-written Matlab code. As most of the analyses focused on narrow band filtered data, to avoid discontinuities (and therefore filter artifacts), we preprocessed continuous data and epoched the data at a later stage in the analysis pipeline. The BrainVision Files were imported into EEG, and independent component analysis (ICA) was applied on the continuous data. ICA components reflecting eye blinks and lateral eye movements were identified and rejected (mean *n* = 3 components removed per participant), using topographic distribution and frequency spectra. Following ICA-based component rejection, data were transformed into Current Source Density estimates, as implemented by Cohen (2014). Data were then filtered, as specified per analysis type (i.e. in 1 Hz steps for alpha, or at the beta harmonic, or the beta band excluding the harmonic), epochs were extracted, and epochs with large residual artifacts were removed, based on visual inspection (mean trials removed = 100, corresponding to 12.5%). Epochs were centered on stimulus onset, from −3 s to +3 s. For the evoked potential analysis, a second round of ICA was applied to the epoched data to remove any residual broadband artifacts (mean *n* = 7 components removed per participant). All data were referenced to the average reference prior to any further analysis.

### E‌EG analyses

EEG analyses focused on 5 occipital/posterior electrodes (Oz, O1, O2, PO7, and PO8), as these posterior electrodes are situated bilaterally across the visual cortex and cover posterior regions with high alpha amplitude.

#### Validation of alpha entrainment response

The alpha entrainment response frequency was calculated using a fast Fourier transform (Matlab’s *fft*), using epoched data from −2,500 ms to stimulus onset. We used this fixed time window to capture the full entrainment period for all participants. The measured individual alpha peak was taken as the peak between 8 and 12 Hz. To test for the accuracy of the measured entrainment response, we then correlated the measured alpha peak with the resting state value (Supplementary Fig. S1-3).

#### Between-group differences in alpha pre-stimulus phase

To validate the between-group phase manipulation, the complex component of the Hilbert transform was used to measure the instantaneous phase of alpha at target onset (converted to radians with Matlab’s *angle.m*). Trials were split according to pre-target interval length (1, 1.5, 2, 2.5 or 3, 3.5 cycles), and the circular average phase (*circ_mean.m* from circstats toolbox) was compared for the timepoint at target onset. Phase clustering within a participant was quantified with Rayleigh’s test for nonuniformity. To assess between-group differences in phase angle, groups were compared with a Watson–Williams test for circular data and the resulting outputs were false discovery rate (FDR)-corrected for multiple comparisons.

#### E‌EG alpha envelope analyses

For the analysis of alpha band amplitude envelope (applied for the entrainment period, pre-stimulus and post-stimulus periods), we used narrowband alpha data that were filtered in a series of 1 Hz steps, from 8 to 12 Hz. All filters were implemented using EEGLab’s eegfiltnew, which uses a Hamming windowed sinc FIR filter. Supplementary Table S1 shows the order and transition bandwidth for each type of filtered data used for analysis. To estimate power change during entrainment, the narrowband data were assigned to “target” or “non-target” bins. The target bin was the bin that contained the entrained frequency for each participant. For example, a participant with an entrained frequency of 9.3 Hz would have a target bin of 9:10 Hz, and the other 3 bins would be labeled as nontarget. For the T-nonMatch group, the nontarget label was subdivided further, to separate out the band containing the participant’s individual measured resting-state frequency.

To calculate the amplitude envelope, the filtered epochs were transformed via the Hilbert transform (using Matlab *hilbert*), and the absolute value of the real component was taken for each time point in the epoch. To account for differences in absolute amplitude between participants, each epoch was normalized (*z*-score) relative to a time window in the pre-entrainment period (−2.8 to −2.7 s before stimulus onset). This method was chosen to emphasize the within-trial effects. We focused on 2 time windows, corresponding to the entrainment and post-target response periods, respectively. The first time window (−1000:−500 ms) was selected to capture a period, during the flicker-induced entrainment, that excluded responses to flicker onset or offset (slowest frequency (125 ms) ^*^ longest cycle duration (3.5 cycles) before target onset). The second time window (400:600 ms) was centered at the trough of the post-target onset decrease in alpha amplitude that is characteristic of the alpha response to a visual stimulus. This window was defined to capture the minimum alpha across participants. The mean latency of alpha amplitude within this window was 522 ms (standard deviation = 68.3 ms) The primary analysis included electrodes across the posterior scalp, to match those used to measure resting state alpha frequency (Oz, O1, O2, PO7, PO8).

To estimate change in the alpha envelope during training, we binned EEG signals across trials into the same 8 blocks (~100 trials per block) as for the behavioral data and tested for main effects of frequency (match/mismatch) and phase (peak/trough) using a linear mixed effects model.

Further, we tested the high beta band (24–30 Hz) as a control frequency, using the same phase and envelope amplitude analyses. This narrow beta band (24–30 Hz) was chosen as it excludes the first harmonic of alpha. To match the frequency resolution for the phase analysis and to test the specificity of phase alignment, we selected the first harmonic of the entrained alpha frequency which falls within the beta band (e.g. for 10 Hz entrainment, the first harmonic was 20 Hz).

#### Event-related potential analysis

We calculated the stimulus-locked evoked response for occipital (O1, O2, Oz) and lateral occipital (PO7, PO8) sensors, in broadband filtered data (1:40 Hz). Data were baseline corrected (−2,800:−2,700 ms prior to target onset) and averaged across the session. For statistical comparison, a 10 ms window around the peak/trough of 3 visual evoked components was used to calculate the component amplitude for each participant. The centers of these windows were aligned to the maximal/minimal values within 3 windows: P1: 70–120 ms (lateral occipital electrodes), N1: 90:200 ms (occipital electrodes), and P2 (lateral occipital electrodes; 250:400 ms). The electrodes sites were selected according to the scalp topography for each component. This procedure allowed us to capture individual differences in component latency and test for between-group differences in latency. Latency was estimated by first averaging data within blocks and across electrode groups, then finding the maximum (positive components) or minimum (negative components) value within the search windows (see above). These per-trial estimates were then averaged across trials to give a mean latency for each block of trials or across the whole session.

## Results

To test the role of individualized alpha entrainment in perceptual learning, we used a visual flicker paradigm that has been shown to result in an increase in alpha power within posterior brain regions (Spaak et al. 2014; Sokoliuk and VanRullen 2016). For each participant, we measured their alpha frequency during a resting-state EEG recording prior to training on the task. We then trained participants on a signal-in-noise task; that is participants were asked to discriminate radial versus concentric Glass patterns embedded in noise. Before stimulus onset, a high contrast square appeared at the center of the screen and flickered for 15 cycles at a rate within the alpha frequency range (8–12 Hz). The target stimulus then appeared at a latency that was scaled by the alpha rate (i.e. at the peak or trough of the flicker-induced oscillation). We manipulated phase of alpha entrainment in 2 frequency-matched groups: (1) Peak-Match (PM): entrainment rate was matched to the individual peak alpha frequency (IAF) and the target stimulus appeared at whole-cycle latencies after entrainment offset and (2) Trough-Match (TM): entrainment rate was set to IAF, but the target appeared at the opposite phase to the Peak-Match group. Data were modeled using a linear mixed effects approach, to account for the unbalanced design. Phase and frequency match were used as independent predictors of learning rate for the 3 experimental groups. A control group, which used the same entrainment parameters as for the Trough-Match group but with an arhythmic visual flicker train, was compared to the best performing group directly.

### Individualized entrainment boosts learning in a phase-dependent manner

**Fig. 2:**
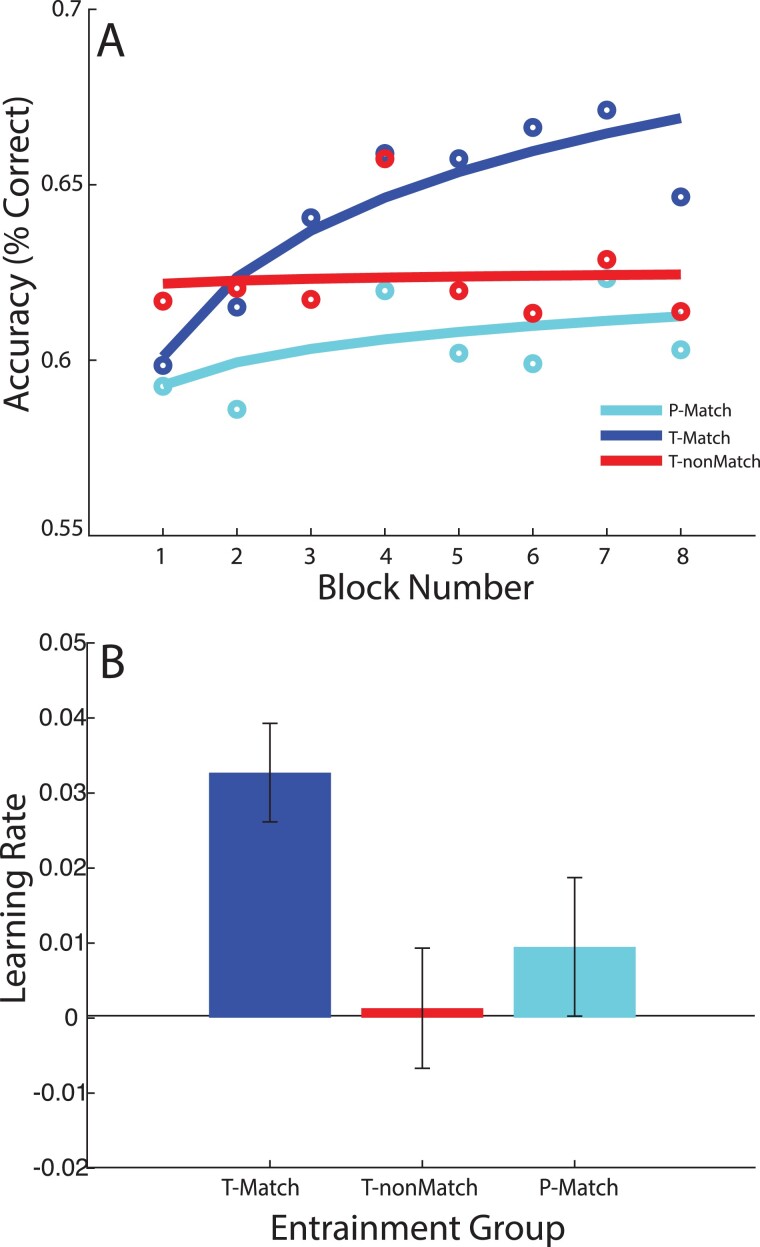
Behavioral performance. A) Performance (% correct) across blocks in Session 1. Open circles indicate mean accuracy (percentage of correct responses) per block of trials (~100 trials per point), for each of the 3 intervention groups (T-Match, T-misMatch, and P-Match). Accuracy data across blocks were fitted with a logarithmic function (solid lines) to estimate learning rate. B) Mean learning rate (i.e. slope of logarithmic fit) across participants per group. Bars show mean learning rate per group, as estimated from fitting the individual accuracy data. Error bars show ±1 SEM.

We found that individualized alpha entrainment with stimuli presented at the trough of the alpha phase accelerated learning (Fig. 2); that is, individuals in the T-Match group showed the fastest learning rates than individuals at the PM and TnM groups (significant main effects of frequency match: [*t*(57) = 2.77, *P* = 0.008; 95% confidence intervals: −5.35/−0.93, estimate = −3.14] and phase: [*t*(57) = 2.05, *P* = 0.045; 95% confidence intervals: 0.11/4.54, estimate = 2.33]). Post hoc tests (least squares difference) showed that learning rate was significantly higher for the TM group compared to the PM (*P* = 0.045) and TnM (*P* = 0.008) groups. In contrast, learning rate did not differ significantly between the PM and TnM groups (*P* = 0.47). These results suggest that individualized alpha entrainment enhances learning, but only when stimuli are presented at the trough rather than the peak of the induced oscillations.

Further, to test whether the T-Match intervention improved learning compared to no-entrainment, we assessed learning in a Control group who received arrhythmic flicker stimulation that was matched in all other parameters to the T-Match group. A one-tailed independent groups *t*-test (Bonferroni corrected for multiple comparisons) showed that there was a significant difference between the Control group and the T-Match group, suggesting the T-Match intervention improved learning (*t*(38) = 2.39, *P* = 0.012; see Supplementary Material Fig. 2.2). Neither the PM nor the TnM group differed from the control group (PM vs. Control: *t*(38) = 0.31, *P* = 0.761; TnM vs. Control: *t*(38) = 1.20, *P* = 0.238). Together, these results suggest that oscillation frequency and phase interact to support learning.

This learning effect could not be attributed to a speed–accuracy trade-off, as reaction times decreased similarly during training across all groups (LME including phase, match and block (first vs. last block); main effect of Block: *t*(57) = 3.00, *P* < 0.004), no significant interaction for either phase (*t*(57) = 1.18, *P* = 0.242) or match (*t*(57) = 1.21,*P* = 0.231). Further, the results could not be attributed to individual variability in performance, as there were no significant differences across groups for starting performance accuracy (no main effect of phase (*t*(57) = 0.270, *P* = 0.788) or frequency match (*t*(57) = 0.561, *P* = 0.577). Finally, we investigated whether the variation in pre-stimulus interval (between 1 and 3.5 cycles) influenced performance in each block. There was no significant main effect of latency on performance accuracy (*F*(2,154) = 0.84, *P* = 0.432) nor a significant block × latency interaction (*F*(2,154) = 0.84, *P* = 0.432). However, the main effect of block remained significant (*F*(6,500) = 5.0, *P* < 0.001), suggesting that the learning differences we observed between groups could not be simply attributed to differences in mean latency.

We next tested whether the entrainment during training had a lasting effect on participant performance. Participants were tested without entrainment or feedback on the signal-in-noise task in a second session (the day after the entrainment session). A LME analysis including phase, frequency match, and day as predictors showed no main effects (*P* > 0.1) and no significant phase × day interaction (*t*(57) = 0.24,*P* = 0.812), or match × day interaction (*t*(57) = 0.81,*P* = 0.422). These results suggest that learning was maintained the day following training. Comparing learning rates across groups in Session 2 did not show any significant differences (LME, main effect of phase: *t*(57) = 0.15, *P* = 0.881, main effect of frequency match: *t*(57) = 1.90, *P* = 0.062), suggesting that differences in learning rate between groups across sessions were due to entrainment during the first session.

### Validation of alpha entrainment

To validate the efficacy of the flicker-induced entrainment, we compared alpha power in the entrainment window (see Methods) across groups (TM, TnM, PM). To capture the entrainment effect, we filtered the data in 1 Hz steps between 8 and 12 Hz and binned the data into “on-target” (i.e. the frequency targeted with entrainment) versus “off-target” (non-entrained frequencies) bands. We reasoned that successful entrainment would result in increased alpha power in the on-target band for frequency-matched groups. In contrast, we did not expect a difference in alpha power during the entrainment window between Peak-Match and Trough-Match groups as the phase manipulation occurred only after the entrainment window. A linear mixed effects model (Phase, Frequency Match, Band) showed a significant main effect of frequency match (*t*(57) = 3.48, *P* = 0.001) and a significant frequency match × band interaction (*t*(57) = 2.05, *P* = 0.045), but no significant main effect of phase (*t*(57) = 0.14, *P* = 0.889). These results suggest that the flicker-induced entrainment increased alpha power specifically at the entrained frequency rather than at immediately adjacent frequencies. Further, this frequency-specific entrainment was stronger for the Matched groups than the nonMatched group.

**Fig. 3:**
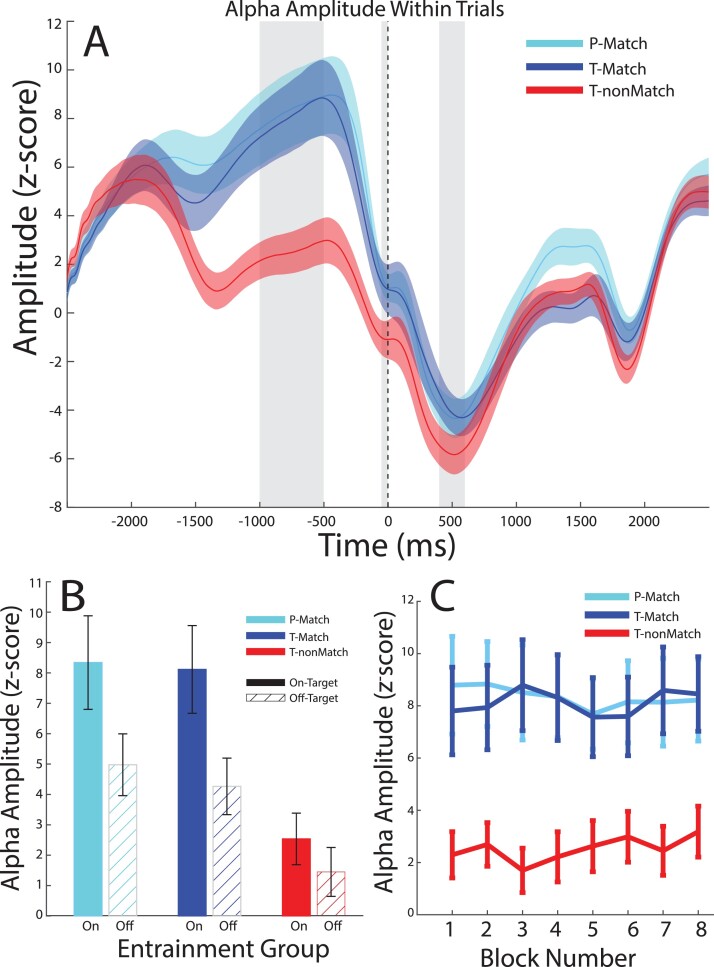
Entrainment effects on alpha power. A) Time series showing amplitude of alpha envelope in the on-target frequencies within the trial epoch. Gray highlighted regions show the 3 time windows of interest for further analysis: entrainment window, pre-stimulus, and post-stimulus (from L-R). Solid lines show the mean per group, shaded regions show ±1 SEM. B) Barplots showing the comparison of mean alpha amplitude within the entrainment window, for on-target (solid color) and off-target (hatched) alpha frequencies across groups. Error bars show ±1 SEM. C) Mean alpha amplitude across participants within the entrainment window for on-target frequencies is shown across blocks for each experimental group. Error bars show ±1 SEM.

Conducting the same analysis in the pre-stimulus window on the rejected ICA components (see Supplementary Material S2) did not show any significant differences (no main effect of match [*t*(57) = 1.15, *P* = 0.256] or phase [*t*(57) = 0.25, *P* = 0.797]) between groups in the on-target alpha power, suggesting that the differences we observed across groups in alpha power could not be due to differences in movement or experimental artifacts across groups. Finally, additional control analyses showed that the entrainment intervention: (i) resulted in a change to alpha amplitude that was specific to the entrainment window (Fig. 3A), (ii) was sustained across the session and apparent from the first block of trials in the entrainment session (Fig. 3C), and (iii) remained appropriately targeted due to the measured stability of the resting state IAF (see Supplementary Methods, Fig. S1).

**Fig. 4:**
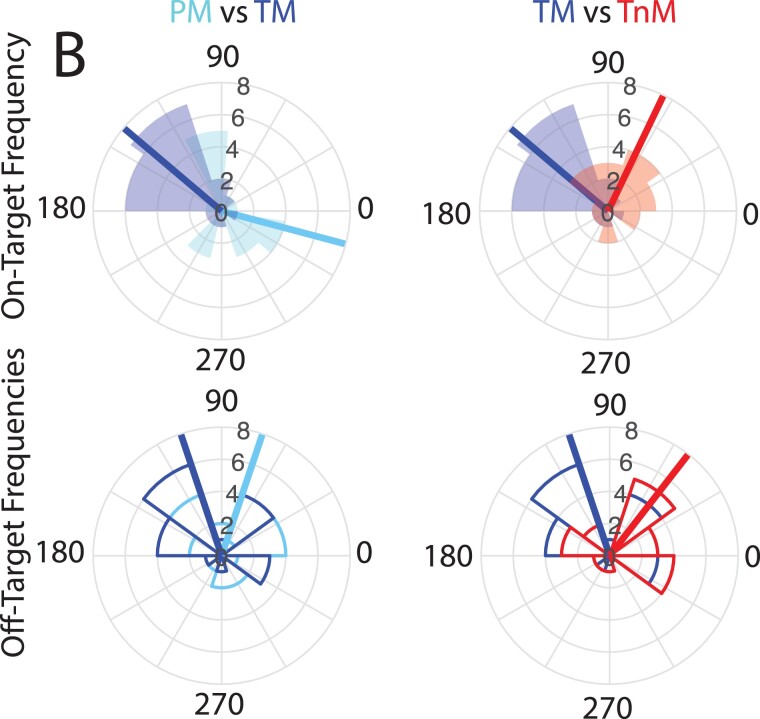
Entrainment effects on alpha phase. Circular mean phase angle for stimulus onset across participants (solid lines) per group; individual values are shown in the corresponding circular histogram. Data are shown for on- versus off-target frequency for 2 comparisons: Trough-Match versus Peak-Match (left column) and Trough-Match versus Trough-nonMatch (right column).

The enhanced alpha amplitude we observed for the frequency-matched, on-target frequency suggests that our targeted entrainment procedure was successful. However, this could reflect power increase from a steady-state visual response. Therefore, we further tested whether the flicker-induced entrainment was successful in manipulating the phase of alpha band activity at stimulus onset. We predicted that alpha band activity for the PM and TM groups would be at opposite phase at the time of stimulus onset, given the scaling of the pre-stimulus interval by alpha frequency. Figure 4A shows the phase clustering statistic for each participant at the time of stimulus onset. Comparing the phase angle of the on- versus off-target alpha at the time of target onset across groups showed a significant difference between PM and TM for the on-target frequency data (Watson–Williams test FDR-corrected: *F*(1,38) = 18.0, *P* < 0.001); in contrast, no phase differences were observed in the off-target frequency data l (*F*(1,38) = 0.81, *P* = 0.50). Further, there was no significant difference between the TM and the TnM group for the on-frequency (*F*(1,38) = 5.09, *P* = 0.060) nor the off-target frequency bands (*F*(1,38) = 0.21, *P* = 0.65). For all groups, entrainment produced significant phase clustering (i.e. nonuniformity) during the entrainment window preceding stimulus onset (Supplementary Fig. S4). Finally, conducting similar phase analyses for a control frequency band (i.e. beta-band) showed no systematic phase alignment effects at stimulus onset (Supplementary Fig. S5), despite some increase in beta power during the entrainment window. This suggests that any temporary increases in beta power that might have occurred during entrainment dissipated quickly and did not carry over into the stimulus onset period. Taken together, these results suggest that individualized (frequency-matched) entrainment results in alpha-band specific phase clustering, consistent with the predicted phase opposition between the TM and PM groups.

### Entrainment modulates early visual processing

**Fig. 5:**
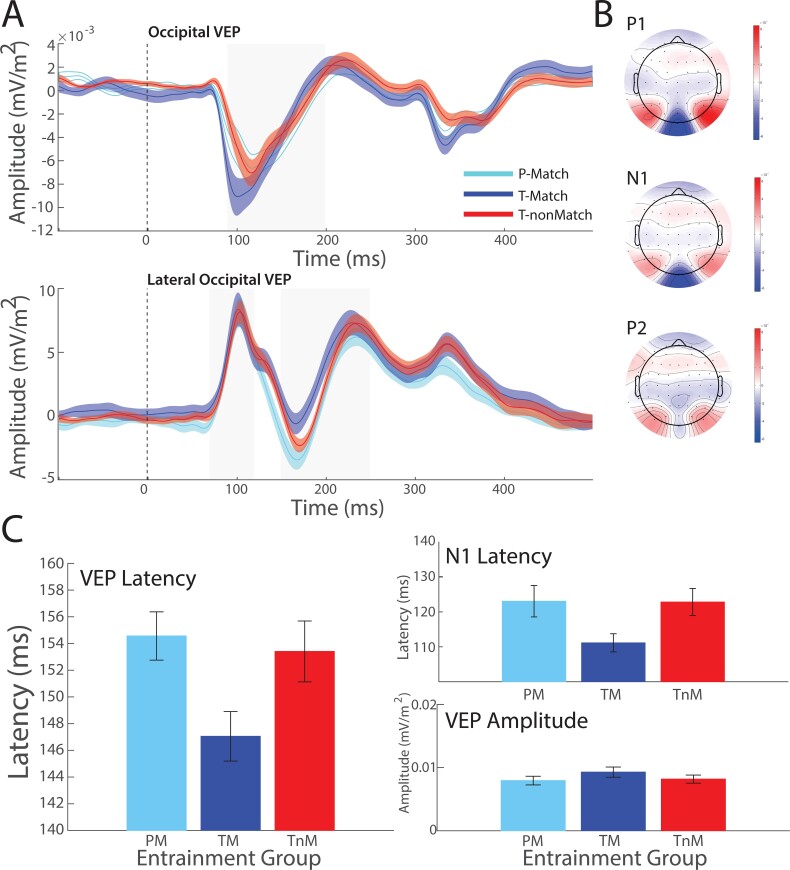
Entrainment effects on visual evoked potentials. A) Posterior evoked potential for all trials, time-locked to the onset of the target stimulus. Top panel shows time series for central occipital electrodes and the gray shaded region indicate the N1 search region. Bottom panel shows the time series for the lateral occipital electrodes and gray shaded areas indicate P1 and P2 search windows. Data are shown for each group; solid line indicates mean amplitude; shaded regions indicate ±1 SEM. B) Topographies for the amplitude of the evoked response averaged within the search windows for each component (P1, N1, and P2 respectively). Black dots indicate electrode sites. C) Left panel: Barplots show mean latency of evoked potential response across participants, following target stimulus onset, for each entrainment group. Top right panel shows the between-group comparison of latency values for the N1 component. Bottom panel shows the mean component amplitude across all components (N1, P1, P2). Error bars indicate SEM across participants.

To investigate whether the flicker-induced entrainment during training altered neural processing in visual cortex that relates to faster learning, we tested for differences in the event-related potential (ERP) (broadband) response to target onset between the entrainment groups. For each participant, we selected 3 components (P1, N1, and P2, see Fig. 5A) of the visual evoked potentials, as these components have previously been linked to alpha oscillations (Klimesch et al. 2004; Gruber et al. 2005) and the processing of Glass pattern stimuli (Ohla et al. 2005; Pei et al. 2005). We compared latency and amplitude of the ERP response in these components in 2 linear mixed models, with factors of group and component. There was no main effect of group and no significant interactions for either amplitude (TM vs. PM: (*t*(57) = 0.37, *P* = 0.714; TnM vs. PM: *t*(57) = 0.14, *P* = 0.887) or latency (TM vs. PM: (*t*(57) = −0.49, *P* = 0.624; TnM vs. PM: *t*(57) = 0.21, *P* = 0.838). There were no significant group × component interactions for either amplitude or latency (*P* > 0.051). However, a priori pairwise comparisons per component based on previous work showing component-specific relationships with alpha oscillations (Klimesch et al. 2004; Gruber et al. 2005; Fellinger et al. 2012) showed that there were main effects of both phase and match for the latency of the N1 (phase: *t*(57) = −2.25, *P* = 0.028; match: *t*(57) = 2.21, *P* = 0.031), reflecting the earlier N1 of the TM group. Taken together, these results suggest that frequency-matched alpha entrainment is associated with the rapid engagement of a neural response associated with visual discrimination (Vogel and Luck 2000).

## Discussion

Using a flicker-induced entrainment paradigm, we demonstrate that individualized alpha entrainment boosts our ability to improve perceptual decisions through training and facilitates early processing in the visual cortex. In particular, we show that brain entrainment accelerates learning when stimulating individuals at their own brain state (i.e. alpha oscillation frequency) in a phase-specific manner (i.e. the target stimulus is aligned to the trough rather than the peak of the entrainment phase). Our findings advance our understanding of the role of brain states in individualized learning for improved cognitive skills in the following main respects.

First, previous work has implicated alpha oscillations in performance in a range of perceptual tasks. Further, recent work has shown that individual alpha peak frequency is a physiologically meaningful measure that mediates the efficacy of flicker entrainment interventions (Haegens et al. 2014; Gulbinaite et al. 2019, 2017). Here, we extend beyond previous work showing that training alters alpha power (Bays et al. 2015), demonstrating a benefit of individualized alpha frequency entrainment on learning ability; that is, entraining at IAF, as measured by resting state EEG, accelerates learning. The visual cortex has been shown to respond to a range of alpha frequencies (Rager and Singer 1998; Herrmann 2001) and local variations in alpha frequency have been reported across different brain regions (Barzegaran et al. 2017) and time (Mierau et al. 2017; Benwell et al. 2019). Here, we provide evidence that entraining the brain at an individual’s dominant frequency at rest boosts learning, while misaligning the entrainment frequency from the IAF (i.e. introducing ±1 Hz frequency shift) reduces the efficacy of the entrainment and any learning benefit. This boost in learning could not be simply due to a rhythmic cue signaling the onset of the target stimulus, as behavioral improvement was specific to the group that received individualized entrainment. It is possible that individualized alpha entrainment increases attentional resources available for stimulus processing resulting in faster learning, consistent with the role of alpha rhythms in the attentional selection of sensory information (Foxe and Snyder 2011; Klimesch 2012; Peylo et al. 2021) and temporal prediction (Hanslmayr et al. 2011; Rohenkohl and Nobre 2011).

Second, we demonstrate that individualized entrainment resulted in increased alpha power during entrainment, validating that flicker-induced stimulation drives alpha oscillations. Alpha power has been suggested to relate to inhibitory processing (Klimesch et al. 2007; Luft et al. 2018) and gain control mechanisms in visual cortex (Jensen and Mazaheri 2010; Van Diepen et al. 2019). It is possible that alpha entrainment in the context of our task (i.e. target identification in clutter) serves to inhibit distracting visual information (Wiesman and Wilson 2019) for efficient target selection and optimized processing through training. Another possibility is that alpha entrainment may modulate attention via an early-stage cortical mechanism that is distinct from the endogenous regulation of alpha activity (Keitel et al. 2019). However, the relationship between alpha power and attention has been controversial (Antonov et al. 2020; Zhigalov and Jensen 2020; Benwell et al. 2022). Our results showing enhanced pre-stimulus alpha, but limited learning for the PM compared to the TM group, suggest that increase in alpha power alone may not be sufficient for learning.

Furthermore, we provide evidence that phase alignment of the target stimulus to the entrainment frequency is critical for faster learning, suggesting that phase gating plays a key role in learning. Phase-linked effects on cognition can be observed in multiple domains (Leong et al. 2014; Leong and Goswami 2015; Clouter et al. 2017; Wang et al. 2018), supporting the critical role of phase in the organization of cortical interactions. It is known that transmission delays may contribute to individual variability in the efficacy of brain stimulation protocols. Further methods that use online monitoring of brain-state (Bergmann 2018) (e.g. phase; (Zrenner et al. 2018; Zarubin et al. 2020) may provide more accurate phase targeting of stimulation delivery. However, as a consistent phase difference was maintained across phase-manipulated Peak and Trough groups, it is unlikely that our results were significantly affected by any between-participant variation in transmission delays that may accumulate. Despite evidence that visual flicker affects phase alignment (Keitel et al. 2019), the role of alpha power versus phase in task-specific behavioral improvement remains debated (Harris et al. 2018; Hülsdünker et al. 2018; Hansen et al. 2019; Zazio et al. 2021). Here, we demonstrate that individualized alpha entrainment boosts learning only when the task-relevant stimulus is aligned to the trough—rather than the peak—of the flicker-induced alpha frequency. This is consistent with previous studies, suggesting that the trough of alpha frequency is associated with stronger disinhibition resulting in increased excitability (Busch et al. 2009; Mathewson et al. 2011, 2010; Fakche et al. 2022). It is possible that individualized entrainment maximizes a form of “pulsed inhibition” (Jensen et al. 2012) that regulates cortical excitability and visual target detection; that is, individualized entrainment provides windows of maximum inhibition (peak) and excitation (trough) to support detection of task-relevant features from noise and visual target identification. Interestingly, we observed behavioral differences despite the fact that the duration of stimulus presentation spans at least one full alpha cycle for all participants. This may suggest that phase is critical for setting the state of the visual system, or that participants did not make use of the whole stimulus presentation window before making their choice (Kiani et al. 2008; Fabre-Thorpe 2011; Tsetsos et al. 2012). However, arbitrating between these possibilities is not possible with the present design and would be an important consideration for future studies.

Finally, we provide evidence that individualized alpha entrainment alters early visual processing (i.e. early visual evoked responses measured with EEG) to support improved perceptual decisions through training. Previous work has shown a relationship between the phase of pre-stimulus alpha (at the peak frequency) and the latency of early visual EEG components (Klimesch et al. 2004; Hülsdünker et al. 2018). Further, training has been shown to alter N1 amplitude and/or latency (Ahmadi et al. 2018; Xi et al. 2020). These results suggest that individualized alpha entrainment enhances attentional selection of target features and optimizes their processing in the visual cortex to support efficient target detection and identification.

In sum, combining flicker-induced entrainment with EEG, we demonstrate that individuals learn faster when learning at their own brain rhythm (i.e. IAF). It is likely that visual flicker induces a widespread entrainment across brain regions. Further work using more spatially precise interventions (e.g. TMS) is needed to determine the role of different brain regions and stimulation protocols (sensory vs. electrical) in brain entrainment. Recent studies suggest that mismatched/non-IAF frequencies may play a functional role in task performance (Nelli et al. 2021; Di Gregorio et al. 2022; Janssens et al. 2022), that is, performance may be optimized by non-IAF stimulation. Here, we did not observe any significant differences between faster vs. slower subgroups, suggesting that these “off-peak” mechanisms may not be critical for learning to extract the relevant information from cluttered visual displays. Our findings shed light into the brain mechanisms that underlie improved learning for perceptual decisions due to entrainment. In particular, individualized entrainment of alpha oscillations may enhance attentional selection and gain control mechanisms in visual cortex to support the detection and identification of targets from background noise. Our findings have strong translational potential for lifelong learning. Investigating the role of intrinsic variations in individual brain states is key for understanding why the same training protocol is effective in some individuals but not others. Our findings provide evidence for the role of individual brain states in learning, proposing that taking into account brain state variability is key for successful training interventions.

## Supplementary Material

MichaelEtAl_SI_accepted_bhac426

## Data Availability

Data for this paper are available from the University of Cambridge repository (Apollo).
