# Supplementary material for: Learning at your brain’s rhythm: individualized entrainment boosts learning for perceptual decisions
Source: Cereb Cortex. 2022 Nov 9;33(9):5382–94. doi: 10.1093/cercor/bhac426 (PMC10152088; doi:10.1093/cercor/bhac426)
Supplement: MichaelEtAl_SI_accepted_bhac426 [file michaeletal_si_accepted_bhac426.docx]

**­Supplementary Material**

**Learning at your brain’s rhythm: individualized entrainment boosts learning for perceptual decisions**

Michael E^1^, Santamaria, L.^1^ Leong V*^1,2,3^, & Kourtzi Z*^1^

**Supplementary Methods**

*Additional analyses of alpha amplitude time series.* To measure whether entrainment produced a sustained response, we compared alpha amplitude across groups in two additional time windows. First, we compared alpha amplitude in the on-target frequency band across groups in a time window preceding stimulus onset (“pre-stimulus window”, 50ms prior to stimulus onset). We did not observe main effects of match (t(57) = 1.95, p=0.056) or phase (t(57) = 0.14, p=0.885) in the pre-stimulus window. Second, there were no significant differences across groups in a post stimulus window (400-600ms post stimulus onset) (match: t(57) = 1.51, p = 0.138; phase: t(57) = 0.166, p=0.869) that has been previously shown to relate to the late stages of perceptual performance (Klimesch et al., 2011). These results suggest that the increase in alpha amplitude was specific to the entrainment window.

*Alpha amplitude across blocks.* Between-group differences in alpha amplitude were present from the first block of trials (see Fig 3C in main text), (significant main effect of phase: t(57) = 2.53, p = 0.014, no main effect of match: t(57) = 0.45, p=0.652). To test for changes in alpha power during training, we compared alpha power across blocks (two-way ANOVA: Block (1-8) x Group). There were no significant changes in first-block alpha power during training, as indicated by a non-significant effect of block (p>0.180)] and no block x phase or block x match interactions were significant (p>0.187). In the post stimulus window, no group showed a significant change in alpha amplitude over time, and there was no significant difference between groups [On-target band, match: t(57) =0.72, p=0.47, phase: t(57) = 0.60, p=0.55 ; Off-target band, match: t(57) = 0.12, p=0.90, phase: t(57) = 0.45, p=0.65]. Therefore, the difference in alpha was present from the first blocks rather than reflecting cumulative effects across the session, suggesting that our alpha entrainment was effective in inducing an immediate change in brain state.

**Supplementary Figures**


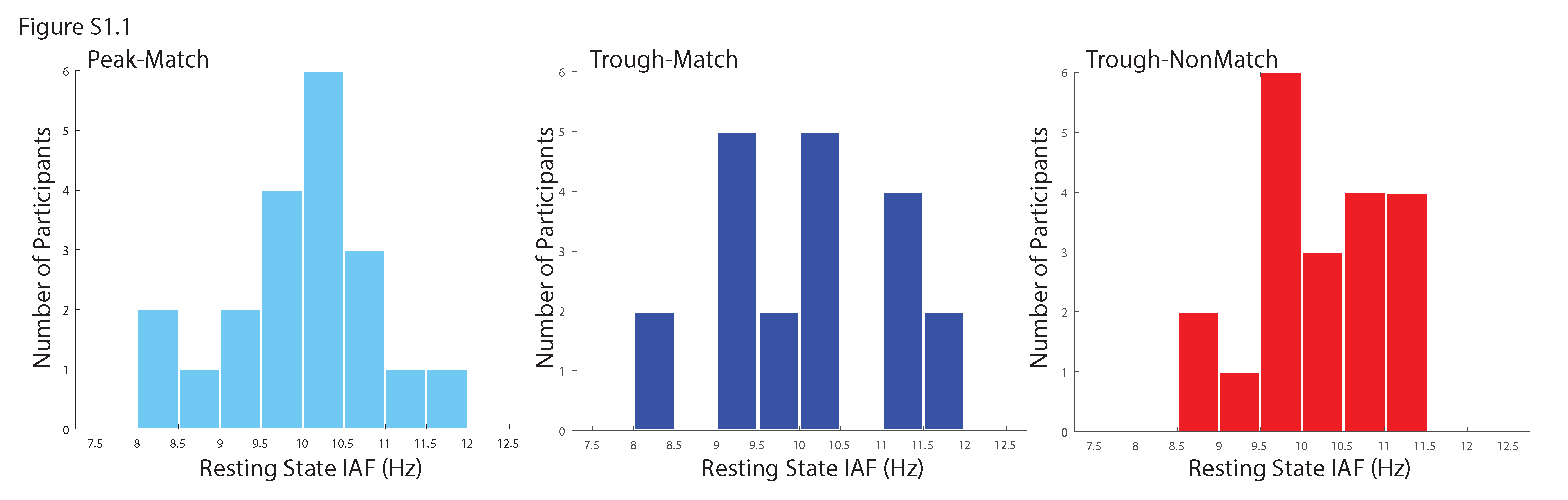


**Supplementary Figure S1: Resting State Measures.**

**Figure S1.1:** Histograms showing the distribution of individual alpha peak frequencies (IAF) calculated from the first EORS for each group (left to right): Peak-Match, Trough-Match and Trough-nonMatch. IAF was measured from a five minute resting state period during which participants were instructed to fixate at a central location and minimize any movements.

The entrainment rate was set relative to the IAF which was calculated from a period of rest at the start of the experiment. The mean difference between the target frequency (IAF) and the entrainment frequency for the matched intervention groups was 0,95 Hz (std = 0.47 Hz). To confirm that IAF was a reliable measure within a session, we took a second resting state measure at the end of the session and compared the IAF from both sessions. Two participants did not complete this second session and therefore we excluded from this analysis. There was a strong correlation between the two measures [r(56) = 0.55, p<0.001, see Figure S1.2 (left subplot)]. Overall, however, there was a significant decrease in alpha frequency over time [t(57) = 2.52, p=0.015], see Figure S1.2 (right subplot). To ensure that the degree of drift did not differ between groups, we compared the mean drift with a one-way ANOVA. We did not find a significant change in alpha for either phase (t(55) = 0.092, p=0.927) or match (t(55) = 0.62, p=0.537) suggesting that differences across groups could not be due to drift in alpha oscillations.


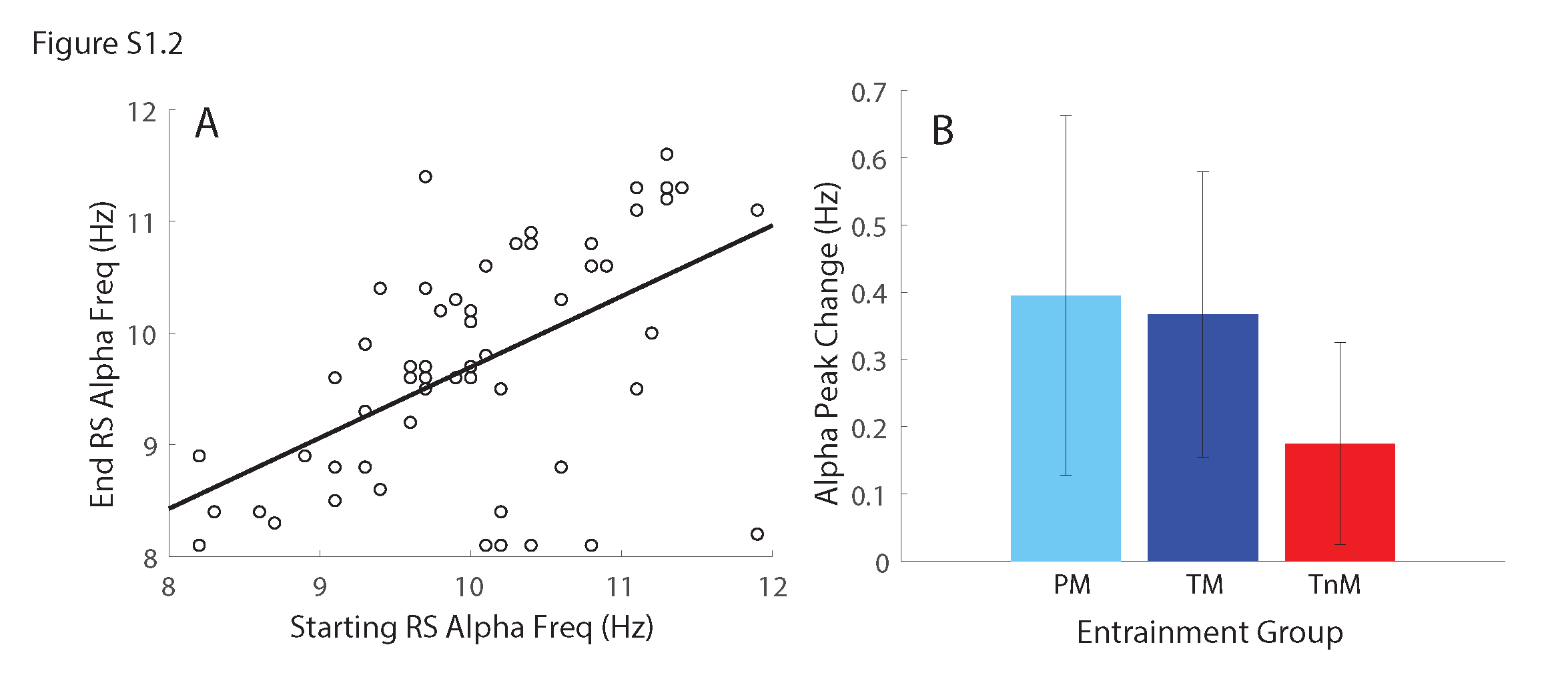


**Figure S1.2. A.** Scatter plot showing the relationship between the measured IAF at the start of the session (x-axis) vs the IAF per participants at the second measurement, which was taken at the end of the experimental session (y-axis). Both measurements were taken while participants were asked to remain still with their eyes fixated at a centrally presented fixation point. The solid line shows the least squares line and each data point corresponds to an individual participant. **B**. Barplot shows the mean change in alpha frequency per group (Peak-Match, Trough-Match and Trough-NonMatch), where a positive value indicates a larger decrease over time. Error bars show +/-1 SEM.


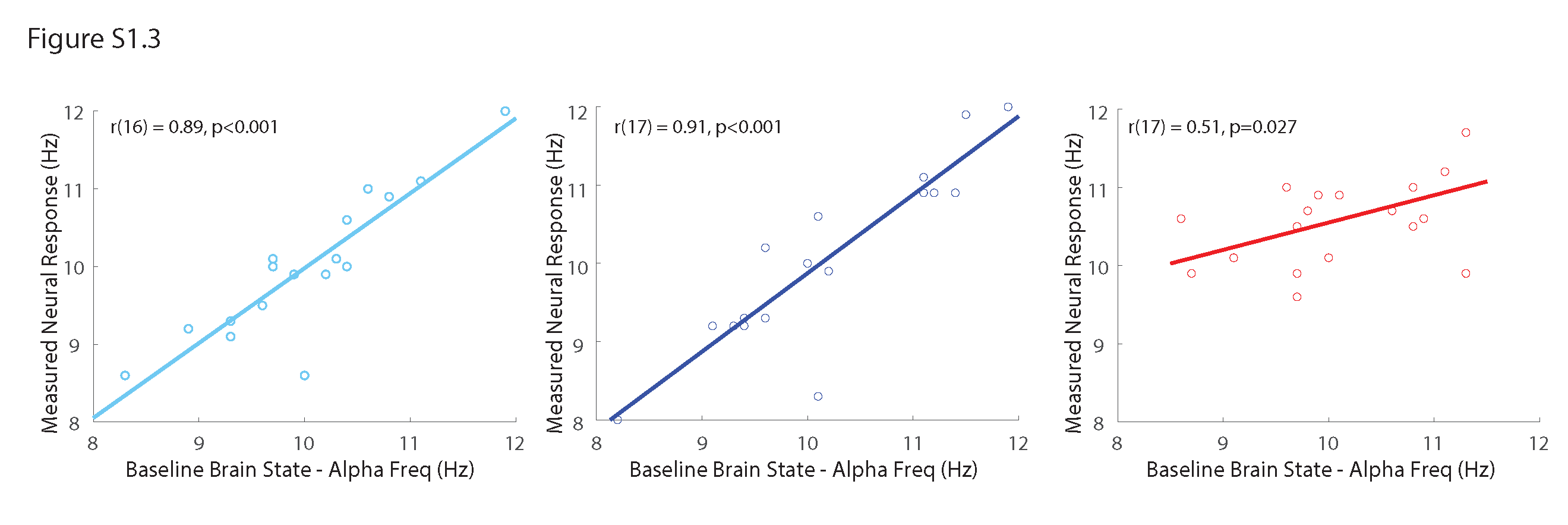


**Figure S1.3.** Scatter plots show the relationship between the IAF (x-axis) and the measured peak alpha frequency during the entrainment period (y-axis). Data are shown per group in each plot: Peak-Match (left), Trough-Match (middle) and Trough-NonMatch (right). Participants with a large (>2Hz) difference between the two measured were excluded from this analysis (PM n = 2, TM n = 1, TnM n =1).


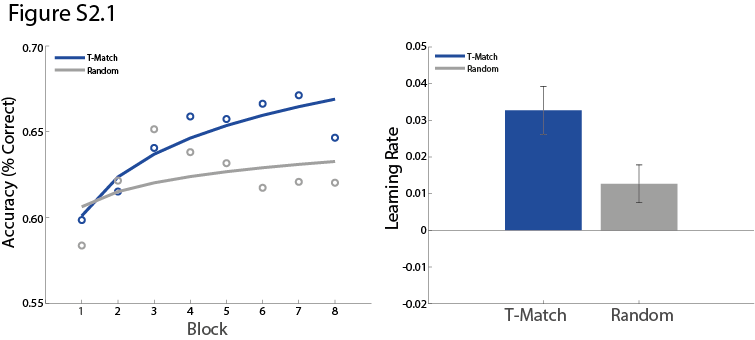


**Supplementary Figure S2.1.** Left panel shows the group average accuracy (% correct) during entrainment, for T-Match (blue) vs the control (grey) group. Open circles show mean accuracy for blocks of trials (~100 trials) and the solid lines are fitted logarithmic functions which capture performance change over time. Right panel shows the group mean learning rates for the T-match group (blue) and the control group (grey, arrhythmic stimulation). Error bars show +/-1 SEM.


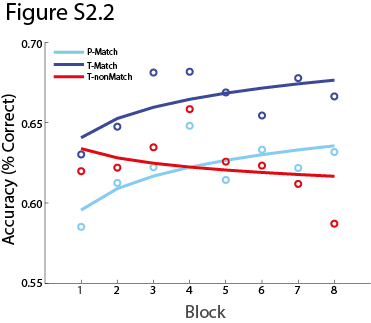


**Supplementary Figure S2.2.** Group average performance, shown per block of trials, for the three experimental groups in the second session. Unfilled circles show the mean performance, and the solid line shows a learning curve (see methods) fitted the group average performance (% correct) across blocks.


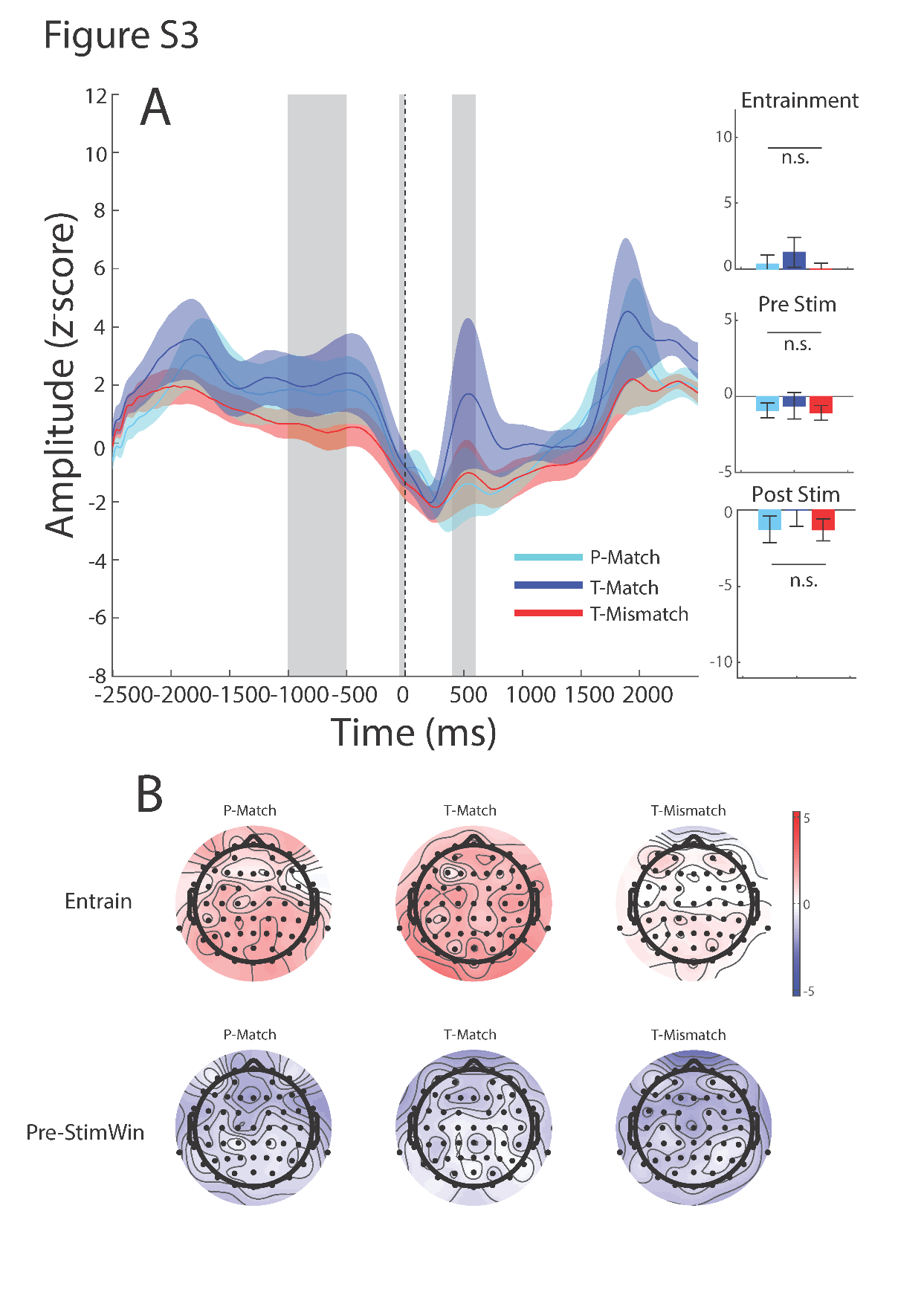


**Supplementary Figure S3. Alpha Power From Rejected ICA Components. A**. Plot shows the mean alpha power per group across the epoch centred at stimulus onset (vertical dashed line). Data here include only the ICA components that were rejected during the preprocessing pipeline. Data were then narrowband filtered in 1Hz steps from 8-12Hz, and alpha power within the sub-band targeted by the entrainment protocol is shown here for each group. The shaded error bars show +/- 1 SEM. Shaded grey regions indicate the windows of interest for the group comparisons (L-R: Entrainment, Pre-Stimulus and Post-Stimulus windows). Bar plots in the right hand panels show the between group comparisons for mean alpha power within each of these three windows of interest. Error bars show +/-1 SEM. n.s. = p>0.05. **B.** Topographies show alpha power within the entrainment (top row) and pre-stimulus (bottom row) windows across all scalp electrodes. Data are shown per group (left to right): Peak-Match, Trough-Match and Trough-NonMatch. Between-groups analysis of alpha amplitude showed no significant between-groups differences, suggesting that the preprocessing was successful in removing only artifactual (non-entrainment) signals.


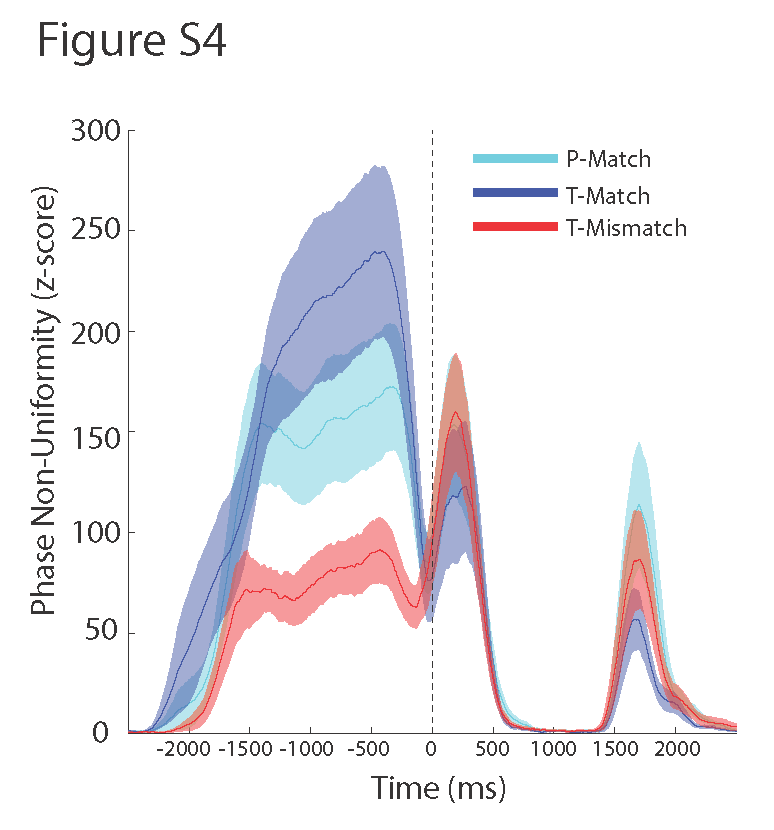


**Supplementary Figure S4. Phase Non-Uniformity in Entrainment-Targeted Band.** To test whether entrainment induced phase-alignment, in addition to the observed increase in power, we calculated the degree of phase non-uniformity throughout the epoch using Rayleigh’s test. All three groups showed a high degree of phase non-uniformity throughout the entrainment period that continued into the post-stimulus window. Plot shows the mean z-score across participants from Rayleigh’s test for non-uniformity (y-axis) across time within an epoch (y-axis). All EEG trials were included in the analysis. Data are plotted relative to stimulus onset (vertical dashed line). For each group, the solid line indicates the mean, and the shaded region shows +/-1 SEM.


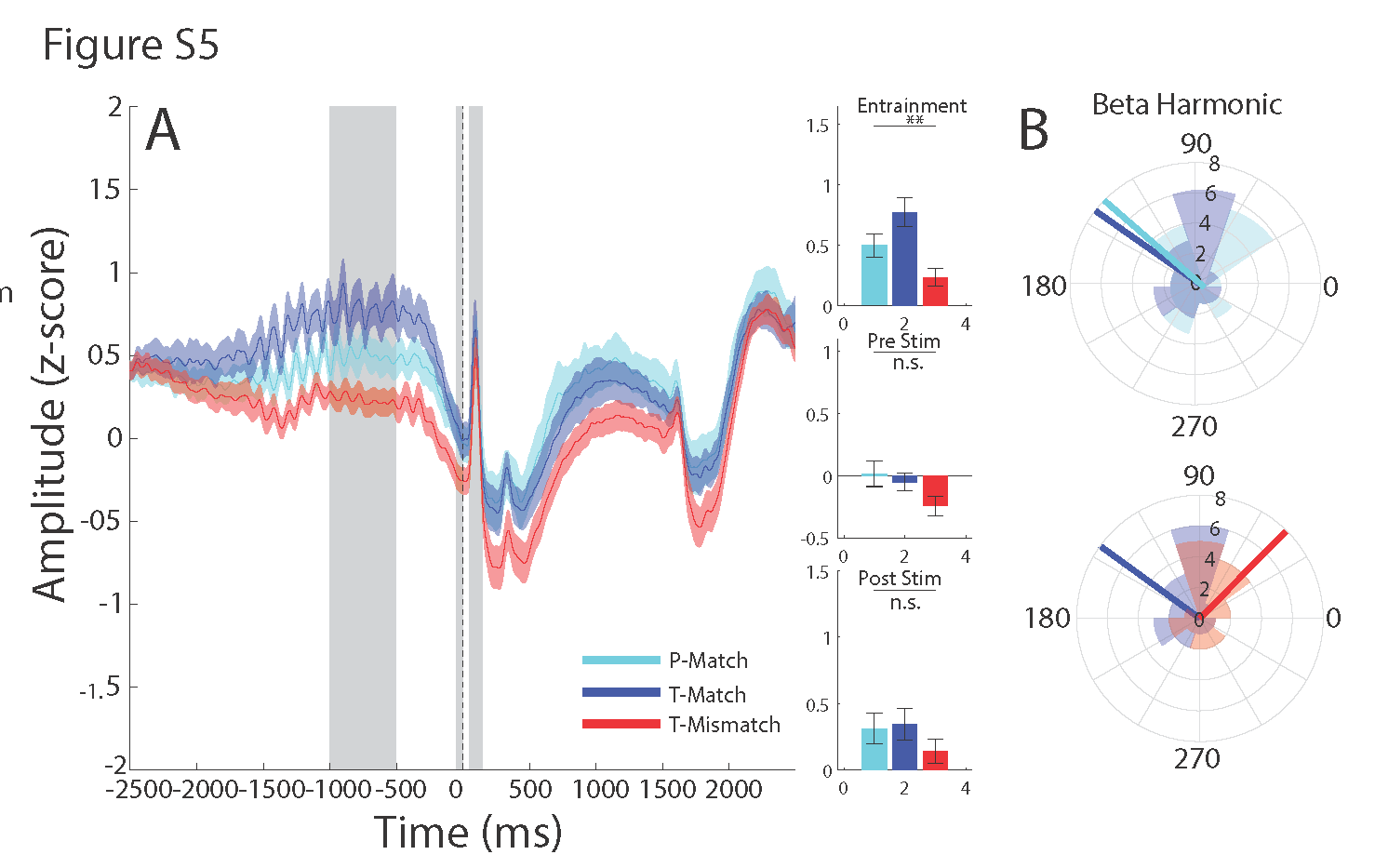
**Supplementary Figure S5. Beta Power Control Analysis A.** Plot shows the mean beta power per group across the epoch centred at stimulus onset (vertical dashed line). The shaded error bars show +/- 1 SEM. Shaded grey regions indicate the windows of interest for the group comparisons (L-R: Entrainment, Pre-Stimulus and Post-Stimulus windows). Bar plots in the right hand panels show the between group comparisons for mean beta power within each of these three windows of interest. Error bars show +/-1 SEM. n.s. = p>0.05. **B.** Estimated beta-band phase at stimulus onset is plotted per group, for two comparison cases. The upper plot shows the comparison between the PM and TM groups and the lower plot shows the comparison between the TM and TnM groups. In both cases, the solid line shows the mean phase per group and the shaded regions show the distribution of individual participant values.

**Supplementary Table 1**

| **Target Frequency (Highpass/Lowpass)** | **Order** | **Transition Bandwidth (Hz)** | **Passband Edge (Hz)** | **Cutoff Frequency (Hz)** |
| --- | --- | --- | --- | --- |
| 8 | 1651 | 2 | 8 | 7 |
| 9 | 1469 | 2.25 | 9 | 7.875/10.125 |
| 10 | 1321 | 2.5 | 10 | 8.75/11.25 |
| 11 | 1201 | 2.75 | 11 | 9.62/12.375 |
| 12 | 1101 | 3 | 12 | 13.5 |
| 24-30 | 551/441 | 6/7.5 | 24/30 | 21/33.75 |
| Entrainment beta harmonic – variable frequency depending on stimulation frequency.  (Min = 16Hz, max = 24Hz) | 827/551 | 4/6 | 16/24 | 14/27 |

Table S1 reports further details on the filter parameters for the “narrowband” alpha analyses and the parallel analyses for other frequency bands. In all cases, filtering was implemented using EEGLab’s eegfiltnew, which uses a Hamming windowed sinc FIR filter. Where two values are give, this reflects the highpass/lowpass filter settings. For the narrow-band alpha analyses, 9, 10 and 11Hz were both lower and upper boundaries and so two cutoff frequencies are listed for these cases respectively.
